# Polymer Grafting and its chemical reactions

**DOI:** 10.3389/fbioe.2022.1044927

**Published:** 2023-01-11

**Authors:** Priyank Purohit, Akanksha Bhatt, Ravi K. Mittal, Magda H. Abdellattif, Thoraya A. Farghaly

**Affiliations:** ^1^ School of Pharmacy, Graphic Era Hill University, Dehradun, India; ^2^ Galgotias College of Pharmacy, Greater Noida, UP, India; ^3^ Chemistry Department, Sciences College, Taif University, Taif, Saudi Arabia; ^4^ Department of Chemistry, Faculty of Applied Science, Umm Al-Qura University, Makkah, Saudi Arabia

**Keywords:** grafting, polymerization, click chemistry, azide-alkyne cycloaddition, drug polymer interaction

## Abstract

Polymer grafting is a technique to improve the morphology, chemical, and physical properties of the polymer. This technique has the potential to improve the existing conduction and properties of polymers other than charge transport; as a result, it enhances the solubility, nano-dimensional morphology, biocompatibility, bio-communication, and other property of parent polymer. A polymer’s physicochemical properties can be modified even further by creating a copolymer with another polymer or by grafting. Here in the various chemical approaches for polymer grafting, like free radical, click reaction, amide formation, and alkylation have been discussed with their importance, moreover the process and its importance are covered comprehensively with their scientific explanation. The present review also covers the effectiveness of the graft-to approaches and its application in various fields, which will give reader a glimpse about polymer grafting and its uses.

## 1 Introduction

The polymer and its chemistry are always been a focus point for the applied chemist despite its origins in the middle of the last century, the polymer system has been found with the numerous applications till so far. (Abourehab et al., 2022; Rehman et al., 2022). The polymer industry is growing as per the growth of population, however, the increasing demand for natural products, which could not be easily met due to limited supplies is a limitation of this industry. The grafting of polymer/copolymers came up with a new hope, which made up of polymer segment branches that are covalently bonded to primary polymer chains. (Ajaz et al., 2022; Awad et al., 2021). The backbone and branches can be composed of different chemical structures or combinations of homo- or copolymers (Vega-Hernández et al., 2021). In the 1980s, synthetic chemists became interested in the possibility of creating new conducting polymers with preferred and enhanced characteristics. (Khedr, 2022). Since then, numerous conducting polymers and their derivatives have been synthesized. Polyacetylene, polythiophene, etc., and many of them are the most prevalent and broadly utilized conducting polymers. The occurrence of conjugated double bonds by the side of the backbone of a conducting polymer is an important and distinct character, which helps to modulate its own property. Conjugation, on either hand, is inadequate for conductive polymerization, however, some other necessity is also required, so that these charge carriers can be injected into the material, like extra electrons or holes. In addition to exposing carriers in the electronic structure, this kind of doping also leads to carrier delocalization along the polymer chain and charge carriers’ mobility that might be extended into three dimensions utilizing inter-chain electron transfer.

A polymer’s physicochemical properties can be modified even further by creating a copolymer with another polymer or by grafting. Grafting polymers are extremely significant because it preserves the extended conjugated structure in the major chain while trying to introduce and incorporate the characteristics of the grafted materials. Grafting has the potential to compensate for and improve able to conduct properties of polymers other than charge transport; as a result, it might be more related to solubility, nano-dimensional morphology, biocompatibility, bio-communication, and so on (Heeger, 2001). Specifically, to meet the criteria for scientific or other benefits the conducting polymers, in particular, requires several other factors than electron delocalization for qualifying the technology or biomedical-based applications. It is difficult to find alternative options to this question so far (Bhattacharya and Misra, 2004; McCullough and Matyjaszewski, 2010).

**FIGURE 1:**
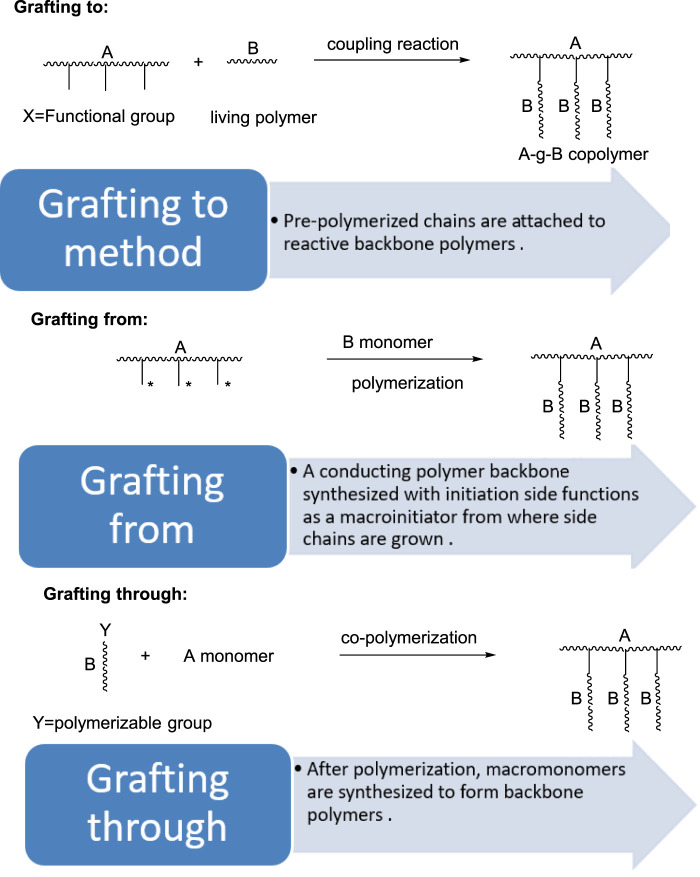
Strategies of polymer grafting: grafting to, grafting from and grafting through.

There are three general methods for synthesizing graft copolymers: 1) “grafting to,” 2) “grafting from,” and 3) “grafting through” it is also summarized in Figure 1 with better representation (Hackett et al., 2018). In summary, the “grafting to” procedure entails attaching pre-polymerized chains to backbone polymers with reactive end-groups (Zdyrko and Luzinov, 2011). The strategy regarding the various polymer grafting was applied to the modification of the polymers as per the given figure below (Figure 1).

### 1.1 Grafting and grafting polymers

The primary goals of surface modification is to improve a surface polymer’s mechanical characteristics, wettability, biocompatibility, and so on.


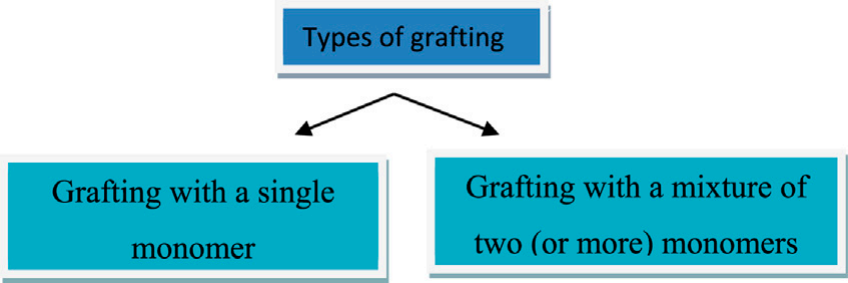


**FIGURE 2:**
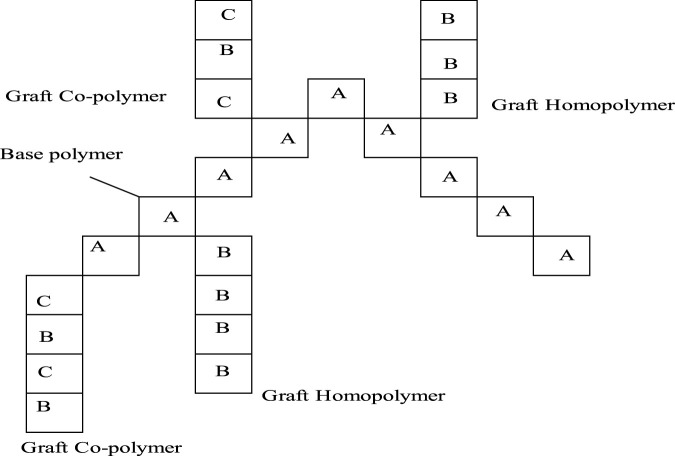
Grafting’ and “grafting polymers.”

Polymer modification is required to provide special properties to the modified material, such as increased thermal stability, multiphase and physical responses, compatibility, flexibility, and rigidity. Through modification, an insoluble polymer becomes soluble, and *vice versa*. It also improves the conditions for polymer processing. The most common polymer modification methods are grafting, cross linking, blending, and composite formations (Figure 2) (Bahulkar et al., 2015).

It is not essential that the polymers obtained to date from various sources have all of the attractive characteristics however, the formulation scientists seeking particular characteristics in polymers may find them lacking in the polymers available. Polymer grafting now plays a major role in the formulation development cycle. Many research laboratories have made substantial progress in chemical modification by presenting innovative reactive functional groups in the chemical structure of polymers, like thio, hydroxyl, amino, and carboxylic acid groups, which indicate possible sites for chemical modification or grafting. In the polymeric age, it is critical to tailor the characteristics of a polymer to specific specifications designed for target applications. Graft polymerization is one of the most appealing and convincing methods, in which one or more polymer side chains are chemically attached to the main polymer chain *via* covalent bonds to alter the rheological properties, hydrophilic ability, polymer charges, molecular chain, aggregation state, and complexing capability of the parent polymer (Thakur et al., 2014; R Bhosale et al., 2015; Slagman et al., 2018).

**FIGURE 3:**
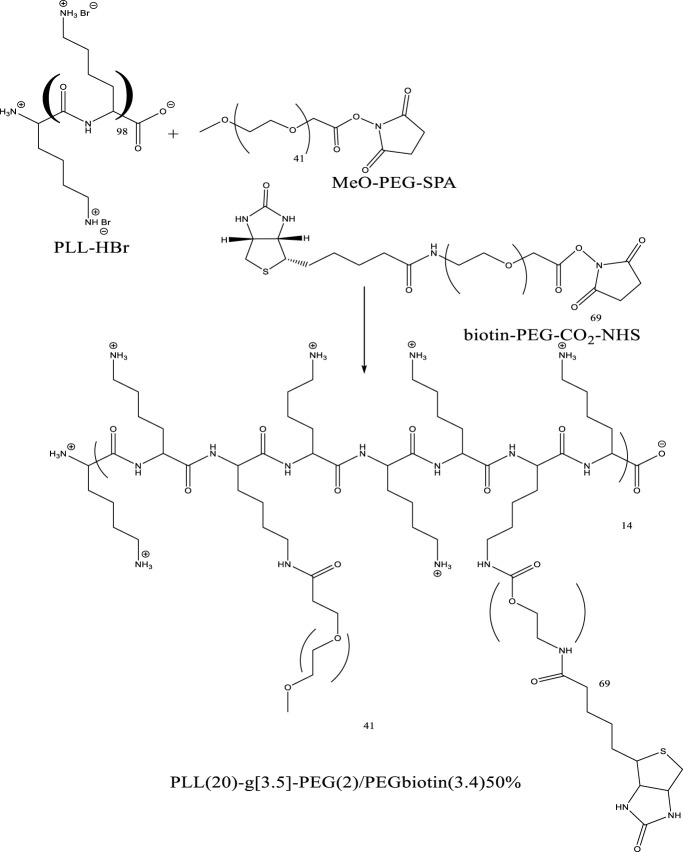
Example of graft copolymer PLL (20)-g[3.5]-PEG (2)/PEGbiotin(3.4)50% from commercially existing constituents.

In the graft-to approach, side chains are connected to a linear backbone *via* a coupling reaction, however the graft-from approach creates side chains from backbone-initiating groups by employing a pre-made backbone polymer as a macroinitiator. Macro monomers with side chains attached are polymerized as part of the graft-through process. The example is depicted below in Figure 3 with the PLL-HBr polymer and its grafting.

Grafting is also classified as types of monomers attached as:

i) Grafting (single monomer): This takes place in a single step.
ii) Grafting (mixture of two (or more) monomers): It occurs when the two monomers are used concurrently or sequentially.

For binary monomer grafting, mosaic grafting has obtained a lot of interest, in this technique, two distinct monomers are grafted next to each other. Grafted co-polymerization is an appealing technique for imbuing a polymer with a range of functional groups. Grafted polymers have a promising future, and their potential is practically limitless. The structure-properties relationship has been a significant research concentrate in polymer grafting. The renewed emphasis on bio-based monomers and biopolymers as a feasible path to lowering (synthetic) polymer waste and disposal problems has energized research efforts aimed at the creation of enhanced materials with substantial biopolymer content (often as backbones) through the grafting of these kinds of materials. The importance of the grafting polymer is reached in the current scenario to the polymer grafting of cellulose, chitosan (Jenkins and Hudson, 2001; Thakur and Thakur, 2014), and polysaccharides (Kaur and Gupta, 2017; Slagman et al., 2018) for the multi-dimensional use as summarized in the below Table 1.

**TABLE 1:** Some examples of grafted polymers.

Backbone	Grafted method	Graft chains	References
Polyethylene	CTP (Chain transfer to polymer) by free-radical Polymerization (FRP) in post-polymerization modification	Vinyl polymers	Hernández‐Ortiz et al. (2017)
Starch	(Mechanically activated) CTP (Chain transfer to polymer) by Free-radical Polymerization (FRP)	Polyacrylamide	Cao et al. (2003)
Polyethylene	CTP (Chain transfer to polymer) by FRP (two-phase system)	Vinyl polymers	Hernández-Ortiz et al. (2019)
Natural rubber latex (core-shell particles)	Chain transfer to polymer (CTP) by emulsion Free-radical Polymerization (FRP)	PMMA	Sirirat et al. (2015)
Poly (propylene glycol)	Chain transfer to polymer (CTP) by Free-radical Polymerization (FRP)	Poly (styrene-co-acrylonitrile	Li et al. (2012)
Polyethylene	Chain transfer to polymer (CTP) by Free-radical Polymerization (FRP) (tubular reactor)	Vinyl polymers	Hernández‐Ortiz et al. (2019)
High-density Polyethylene (HDPE)	Melt free-radical grafting	GMA	Saeb et al. (2017)
Various surfaces	Surface-initiated controlled radical polymerization.	Polymers from 2-methacryloyloxythyl phosphorylcholine (MPC), methyl acrylate, acrylamide, and N-isopropyl acrylamide	Zhou et al. (2012)

## 2 Techniques of polymer grafting

**FIGURE 4:**
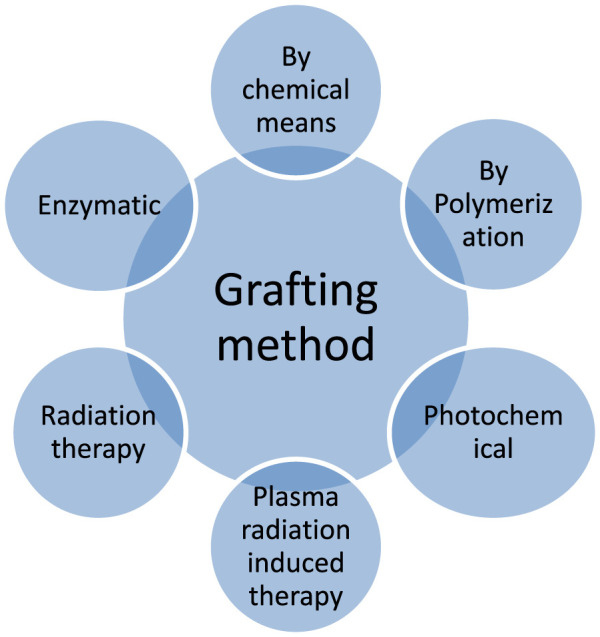
Overview of grafting technique.

Several factors influence the polymer grafting techniques and its reactions involve in, like the chemical nature of the system’s components (backbone, monomer, initiator, and solvent) and their relations. Numerous different factors of polymer grafting must be considered, like temperature and additive use (Sosnik et al., 2011). The synthetic route of graft polymerization is given below in Figure 4 with the activators include a variety of exciting and versatile polymer modification routes.

### 2.1 Grafting through chemical

The chemical grafting is classified into two main routes: free radical and ionic initiator grafting The function of the initiator is critical because it explains the route of the grafting method.

#### 2.1.1 Free-radical grafting

In this kind of chemical reactions, the generation of free radicals take place, which transferred to the substrate for the reaction with monomers to form the graft copolymers. For the polymer grafting, various polymerization methods have been used, but free radical processes (e.g., FRP, RDRP, and REX) are the most efficient because of their flexibility in working with distinct chemical groups and tolerance to impurities. The polymer can be formed in this grafting by irradiating macromolecules, which causes homolytic fission. The longevity of free radicals is determined by the behavior of the polymer backbone which is essential parameter to form the stable graft polymers. Several factors influence the polymer grafting by FRP and other reactions, including the chemical nature of the system’s components (backbone, monomer, initiator, and solvent) and their interactions, e.g., (Sosnik et al., 2011).

$$Fe2++H_{2}O_{2}\to Fe3++OH-+O\dot{H}$$

$$Fe2++-O_{3}S-OO-{SO}_{3}^{-}\to Fe3+{SO}_{4}^{2-}+{SO}_{4}^{-}.$$

**FIGURE 5:**
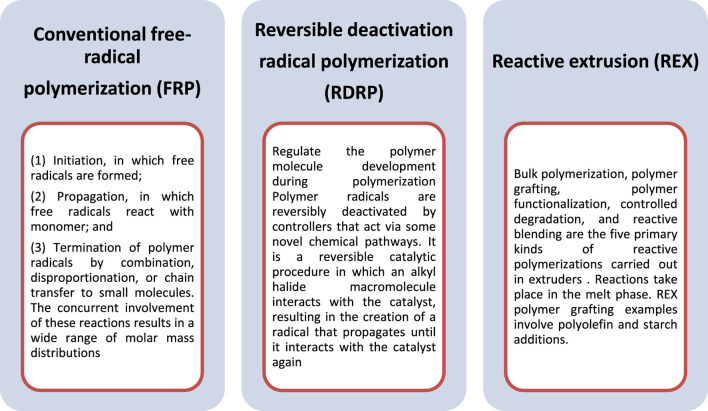
Types of Free-radical polymerization methods.

Polymer grafting has been accomplished using a variety of polymerization methods, but free radical techniques (e.g., FRP, RDRP, and REX) are generally the most efficient method due to their versatility in working with distinct chemical groups and tolerance to impurities. This method continues to produce roughly 60% of all accessible polymers (Šebenik, 1998). The various methods are categorized here the below in Figure 5.

**FIGURE 6:**
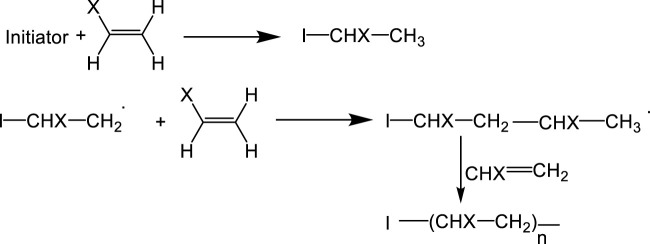
Example of free radical polymerization.

The initiators, which is known as beginner of polymerization, generates free radicals, which are transferred to the substrate and act in response to the monomer to make the graft copolymers (Figure 6). When free radicals react with a monomer, they produce a new radical that can initiate chain growth of polymerization (Sumerlin et al., 2005). Benzoyl peroxide (BPO) and 2,2′-azo-bis-isobutyrylnitrile are two common initiators (AIBN) for the free radical based polymerization. The initiators are sometimes incorrectly referred to as catalysts, however they inspires the reaction, whereas catalysts are regenerated later than the reaction is completed.

Where, X is a substituent that could be any of C_6_H_5_, Cl, Br, OCOCH_3_, COOR, or H. Di-substituted monomers such as vinylidene chloride and methyl methacrylate are also included in the mechanism.

## 3 Click chemistry

**FIGURE 7:**
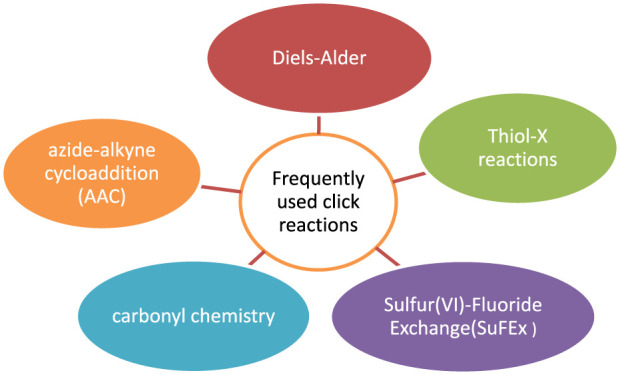
Usage of click reactions for polymerization and polymer modification.

The formation of 1,2,3-triazole by the 1,3-dipolar cycloaddition of an azide and an alkyne is known as click chemistry. The ease of its preparation and purification makes it appropriate for the broad variety of applications even for new development because of the speed and acceptability of reaction. Polymeric soft materials with precise architectural and functional control can be made using click chemistry, a well-known powerful method. It is great that the efficiency of click chemistry could be combined with the chain-end functionality control of live free radical polymerization (Kolb and Sharpless, 2003). In 2001, the Sharpless and colleagues modulated the reactions condition drastically to change the product yield and its acceptability as a “click” reaction in the scientific arena. Since then, other click chemistries have been developed in polymer science (Figure 7) to produce macromolecular materials with specific properties for a variety of uses. As more and more reactions are discovered, click chemistry’s scope expands (Zhang et al., 2018).

### 3.1 Click chemistry in polymerization and polymer modification

This section highlights the range of click reactions, along with their benefits and drawbacks, to help the reader to choose the best click reactions. The most commonly used click reactions for polymerization and polymer modification are azide-alkyne cycloaddition (AAC), Diels-Alder, thiol-X reactions, carbonylbased addition, and SuFEx. More specifically, advances in click chemistry enables the synthesis of copolymers with novel architectures, such as heterolayer dendrimers, dendron-polymer conjugates, and nano-constructs, as well as conjugation with a broader range of biopolymers and living systems in complex media. It is also reported that functional cyclic backbone, and cyclic-graft polymers can be grafted by linear polymers (Opsteen and van Hest, 2005).

Dendrimer a 3D globular topological polymers are made up of three major components: a) an outer group; b) branched cells; and c) a focal point (Li et al., 2017; Zhang et al., 2017). They have a lot of internal cavities, branched structure, and a greater degree of geometrical symmetry with carefully controlled processing. Dendrimers exhibits a number of architecture-induced phenomena, such as the branched cell symmetrical impact, architectural amplification, and nanoscale features (Zeng et al., 2019; Mignani et al., 2020). In addition, the dendrimers have a variety of structural components, among them he five commonly used dendrimer groups include poly (lysine) poly(amidoamine), poly(propylenimine), phosphorus, and carbosilane (Dzmitruk et al., 2018).

Click chemistry has been successfully accepted for the synthesis of dendrimers by using convergent and divergent growth strategies. The first convergent technique was used by Wu et al. (2004) with AB2-monomers, where the A-functionality was a chloromethyl and the B2-functionalities comprised acetylenes. Azide moiety was easily introduced by the substitution of the chloride atom for enabling click-reaction, and following coupling step. Eventually the fourth generation dendrons were linked in 1-4 di and 1,2, 3 triazole to build a range of various core molecules to add on the properties of the parent polymer. The main accomplishment of this research was to show how useful Click chemistry was and how much effectiveness could be obtained during a typically challenging synthetic approach. Joralemon et al. (2005) remarked on the divergent technique for Frechet-type dendrimers utilizing Click chemistry, leading in azide- or acetylene-terminated dendrimers. Hawker et al. previously investigated the application of thiol-ene Click chemistry, for the divergent technique of poly(thioether) dendrimers. Even more environment preferable, the synthesis was completed without the assistance of a metal catalyst and under moderate reaction conditions (Killops et al., 2008).

#### 3.1.1 Azide-alkyne cycloaddition

**FIGURE 8:**
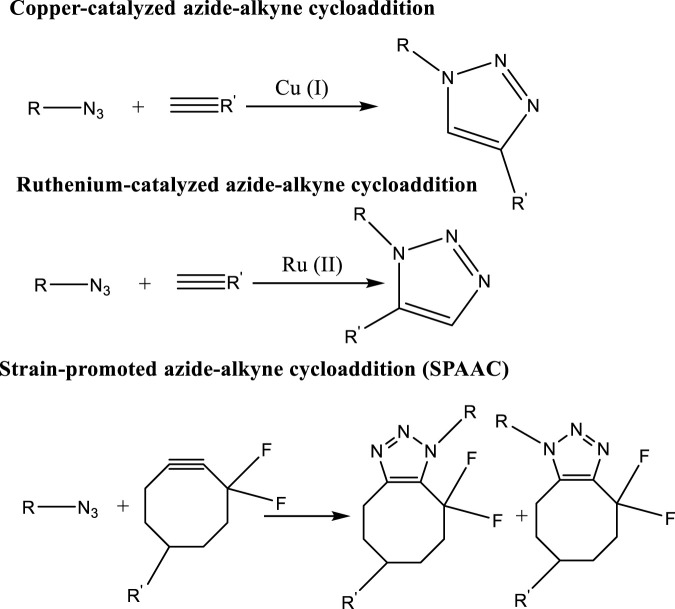
Example of click reactions and their use.

Polymer-polymer conjugation, or CuAAC (3-4), is a promising approach to addressing some of regioselective issues over the conventional polymerization methods. For the purpose of providing a metal- and catalyst-free alternative to standard Cu and Ru metal mediated Azide-alkyne Cycloaddition (AAC), and strain-promoted alkyne-azide cycloaddition (SPAAC) were developed as depicted in the below given Figure 8 (Lutz, 2008; Sletten and Bertozzi, 2011; Li and Cheng, 2013).

#### 3.1.2 1,3-dipolar cycloaddition

**FIGURE 9:**
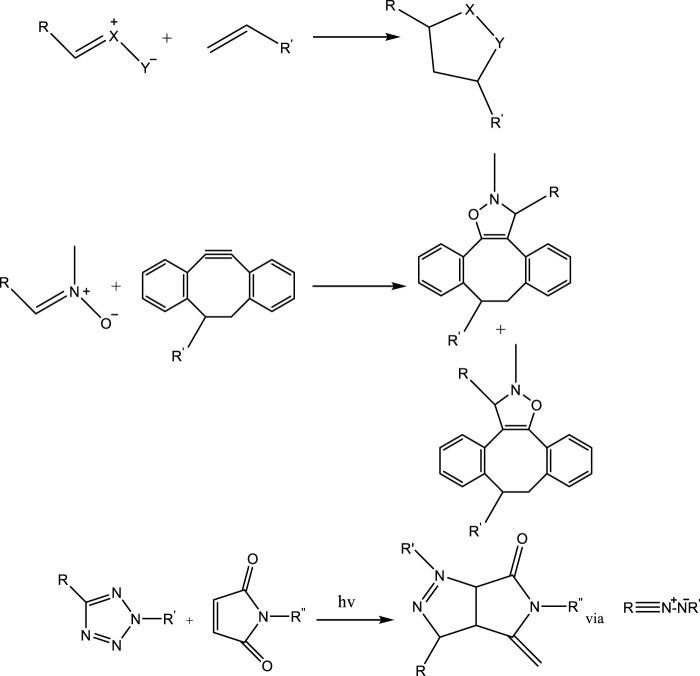
One to three dipolar cycloaddition reactions.

The CuAAC and SPAAC have been widely used method for the research purpose for developing a new tool for the 1,3-dipolar cycloadditions (Figure 9), moreover that also meet the click chemistry specifications. Reactions relying on the intermediate’s nitrile oxide, nitrone, nitrile imine, and nitrile ylide are becoming increasingly popular for their advantageous reactivity.

#### 3.1.3 Nitrile oxide-alkyne and alkyne-nitrone cycloaddition

**FIGURE 10:**
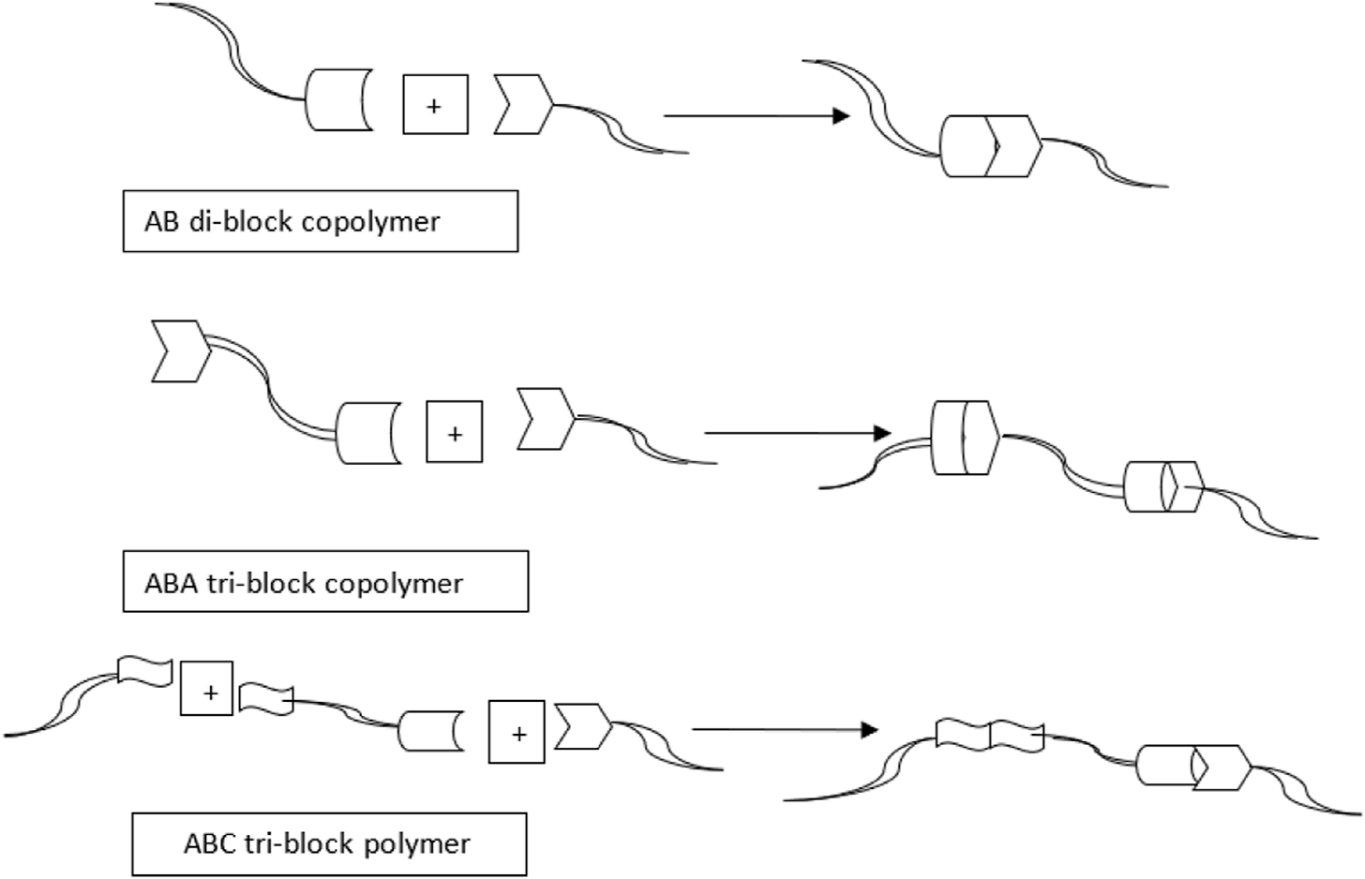
General example of the use of click chemistry (Tasdelen et al., 2011)

Nitrile oxides are highly reactive species that are frequently produced *in situ* from hydroxamoyl chloride for subsequent reactions like alkyne-nitrone cycloaddition (ANC). The nitrile oxide alkyne cycloaddition reaction is a highly efficient method for clicking the functionalization of polymers with a variety of alkynes, particularly strained alkynes. Strain-promoted alkyne-nitrone cycloaddition (SPANC) has proven particularly useful as an orthogonal click reaction for bioconjugation (Figure 10) (McKay et al., 2010; Ning et al., 2010; Geng et al., 2021).

In the production of complex polymer structures, [(multi)block copolymers, star and branching polymers, and cyclic polymers], the chain end functionalization is an essential first step, which proceeds two distinct ways like i) post-polymerization modification and ii) polymerization with a clickable initiator to exhibit functional chain ends. Polymerization using clickable initiators is the frequently applicable procedure for addressing post-polymerization modification issues such as intra- or inter-chain side reactions and incomplete chain end conversion (Mansfeld et al., 2010; Feng et al., 2011; Chen et al., 2016a).

### 3.2 Graft copolymer using click chemistry

**FIGURE 11:**
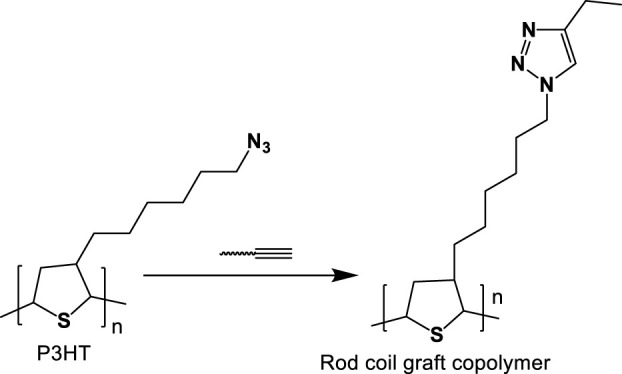
General procedure for constructing poly(3-hexylthiophene-)-related rod-coil graft copolymer through CuAAC.

The graft copolymers have a linear backbone with randomized spaced branches. The formation of well-known graft copolymers with preferred functional groups, controlled lengths, and backbone and side chain contents is highly popular. Graft copolymer synthetic methodologies are divided into three categories graft-to, graft-from, and graft-through. These strategies maximize grafting effectiveness while minimizing un-reacted side chains. Matyjaszewski and others were the first to investigate the use of CuAAC in the production of graft copolymers as depicted below in Figure 11. In this case, CuAAC was prepared using **PHEMA (Poly(2-hydroxyethyl methacrylate))** with alkynyl side groups in a variety of azido-terminated polymers such as PEO-N3, PS-N3, PnBA-N3, and PS-b-PnBAN3. Grafting densities were below 50% for PS, PnBA, and PS-b-PnBA due to stearic hindrance of the connected side chains, so although grafting densities for less bulky PEO side chains attained up to 88%, (Gao and Matyjaszewski, 2007; You et al., 2014).

## 4 Other methods are

### 4.1 Bromination

**FIGURE 12:**
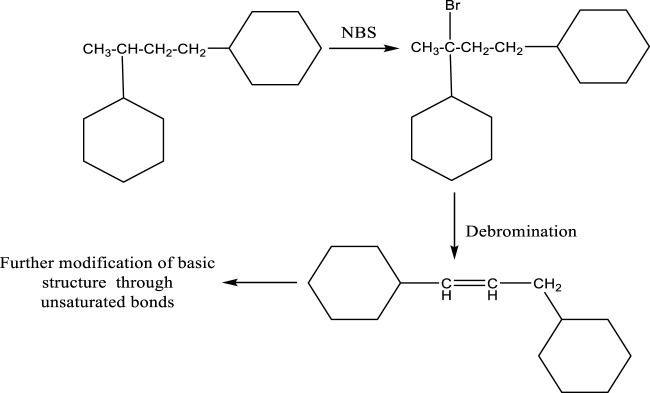
Polymerization/polymer grafting through bromination.

This technique was used to make sure that substitution happened preferentially in the molecule’s unsaturated part, as polystyrene bromination, was prepared by thermally polymerizing styrene at 70°C and was thought to have little or no branching. The limitation of technique is the termination of growing chains would result in cross-linking and the formation of an insoluble network all through polymerization, it is avoided only by the radical termination method (Figure 12), where the primarily disproportionation is essential. The below given picture is the classical example of bromination to get the unsaturated bond for further polymerization.

The chain length and branching are controlled by the rate of reaction and other kinetic parameters, which usually can be found in the polymerization of styrene (Carlin and Shakespeare, 1946; Jones, 1956).

### 4.2 Esterification

Esterification is the reactions of acid and alcohols in presence of acid, where the nucleophilic alcohol reacts with the electron deficient carbonyl and forms the ester bond. These bonding gives the polymer a new property without destroying intrinsic property. The esterification of cellulose with nitric acid in the presence of sulfuric acid, phosphoric acid, or acetic acid results in the formation of cellulose nitrate has been reported with the beneficial activity, moreover the cellulose acetate, cellulose acetate propionate, and cellulose acetate butyrate have been found economically valuable cellulose esters (Klemm et al., 1997).

### 4.3 Etherification

This is the primary method of chemically altering the structure of cellulose and cellulose based material like methylcellulose, carboxymethyl cellulose, and hydroxyalkyl celluloses, they are examples of economically valuable cellulose ethers. Another method for modifying the structure of cellulose is to react it with bi- or poly-functional compounds to produce cross-linked or resinification products in the cellulose matrix. These methodologies can stabilize the cellulose structure and give it crease resistance (or “durable press” properties) (Stevens, 1990; Freire et al., 2006).

### 4.4 Chain transfer

**FIGURE 13:**
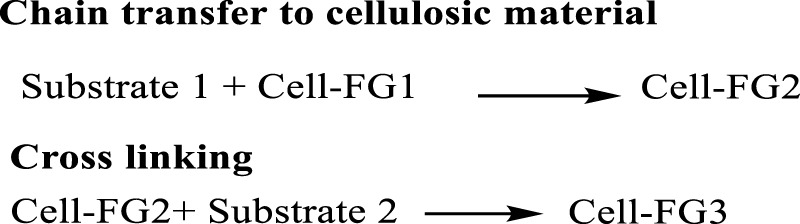
Summary of chain transfer for cellulose grafting (FG, Functional Group, Cell: Cellulose).

The chain transfer reaction is widely used in the cellulose grafting, however the termination of the reaction is the matter of concern, in the case of cellulose chain transfer, the polymerization is stopped by removing hydrogen atoms from the cellulose molecule, which causes radical formation from the cellulose backbone. In various cases the thiol or xanthate ester groups are introduced, because purely radical transfer graft copolymerization does not obtain with the higher graft yields, however the substances with higher chain transfer action, like thiol groups, can be introduced into the cellulose molecules before grafting. The generation of the activating species on the swollen cellulose substrate backbone occurs during potassium persulfate initiation. Cotton cellulose, for the example given below in Figure 13, was modified by Ghosh and Das by grafting acrylic acid in the presence of potassium persulfate as a free radical initiator (Chaudhuri and Hermans, 1961; Pulat and Isakoca, 2006).

### 4.5 Ionic and ring opening graft polymerization

**FIGURE 14:**
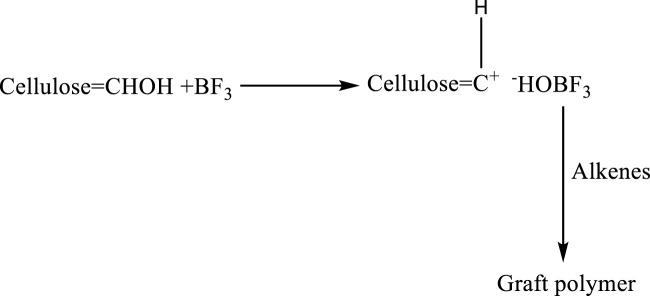
Cationic graft copolymerization Mechanism of cellulose with isobutylene.

The ionic and ring opening graft polymerization is less explored because of the stringent reaction conditions by ionic polymerizations. Rausing and Sunner investigated the cation-mediated grafting of isobutylene and Alpha methyl styrene onto a cellulosic substrate. Polymerization of anionic grafts as anionic graft polymerization was used to create acrylonitrile, methacrylonitrile, and methyl methacrylate graft polymers on cellulose as given in Figure 14 (Bhattacharyya and Maldas, 1984; Perrier and Takolpuckdee, 2005).

### 4.6 Ring-opening polymerization from cellulose fibers

**FIGURE 15:**
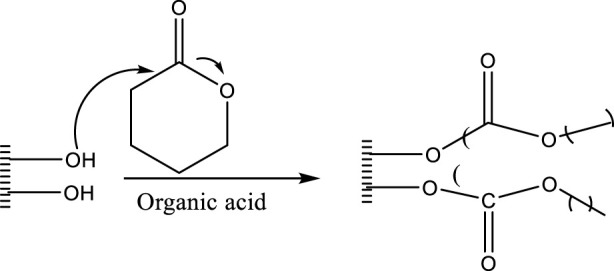
Organic acid-catalyzed ROP from cellulose fiber.

Hafren and Cordova reported (Figure 15) the first direct organic acid-catalyzed ring opening polymerization of cyclic monomers like epsilon-caprolactone (epsilon-CL) using solid cotton and paper cellulose as initiators. The amount of free initiator added to the amount of monomer determined the degree of graft polymerization (Hafrén and Córdova, 2005).

## 5 Application of graft polymerization

### 5.1 In pharmaceutical field

There are several types of drug delivery systems that make use of a broad range of grafted natural polysaccharides as depicted in Table 2. Natural polysaccharides and their derivatives have been used to regulate medication distribution in the pharmaceutical and biomedical industries. The primary benefits of a CDDS are the maintenance of an optimal concentration, often for long periods, the improvement of the action of accountable drugs because of their resistance to the hostile environment, and the diminution of undesirable action because of the high initial blood concentration (Singh et al., 2000).

**TABLE 2:** Examples of polymer grafting in pharmaceutical field.

Example	References
Singh et al. also observed that in colon targeted drug delivery, the drug tetracycline hydrochloride was released from polymethacrylamide polymeric networks modified with N, NMBA Am as a crosslinker and ammonium persulfate (APS) as an initiator.	Dholakia et al. (2011)
The antibacterial effect of chitosan and a grafted sample was discovered by utilizing gram + and gram ^_^ bacteria. Grafted components significantly enhance anti-bacterial action	Koroskenyi and McCarthy (2002)
Radicals are controlled by the action of antioxidants like vitamins A, C, and E1, which help in the donation of electrons to the radicals. (Figure 16)	Iskandar (2011)

(In the synthetic method explained in Figure 16, the ascorbic acid/hydrogen peroxide redox pair, a biocompatible and water-soluble system, was utilized as radical initiators: the hydroxyl radical, which initiates the reaction, is developed by the oxidation of A, where A is a Hydroxy bearing substrate, by H_2_O_2_ within the ascorbate radical.)

**FIGURE 16:**
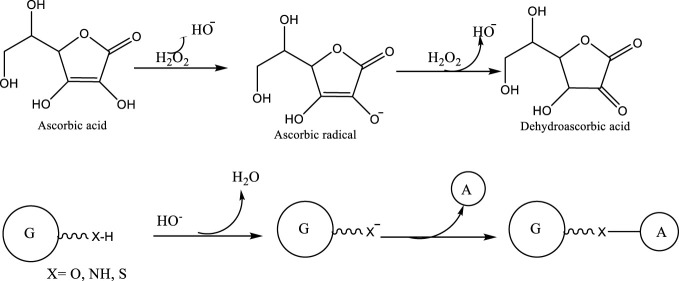
Grafting of gelatin (G) with antioxidant (Ascorbic acid) molecules.

### 5.2 Plastic industry

Polymers by now replaced many conventional techniques, including metal, wood, ceramic, and glass in the majority of earlier applications as prior. It was predicted that combining two bio-degradable initiator supplies, like starch and natural rubber latex, with polymethyl methacrylate would result in renewable degradable plastic (Dilara and Briassoulis, 2000).

### 5.3 Textile industry

The flocculation effectiveness of the grafted polymer was initiated to the best at pH 4.0 for SS (suspended solid) removal and pH 7.0 for TDS removal when used at the optimum dose. At acidic pH (4.0), neutral pH (7.0), and alkaline pH (9.2), maximum SS removal (94.4%) occurs over 1 hour of contact time, with only 10.5% and 44.3% SS excluded. At neutral pH, the maximum TDS removal (80.6%) was noticed. (Kumar et al., 2017).

### 5.4 Other

The polymer grafting techniques has reached to the membrane separation science, conducting polymers and hydrogels (Alves and Mano, 2008). The physicochemical property of chitosan was modulated by the grafting polymer technique, by the derivatization of substituent carboxyl groups, which demonstrates zwitterionic properties such as good flocculation capabilities in both acidic and basic mediums, despite the fact that chitosan is an efficacious flocculating material only within an acid medium to accomplish (Dailey et al., 2005; Sakhare and Rajput, 2017). Dailey et al. (2005) exhibited that grafting of PVA polymer with PLGA to increase the material’s ability to transport biomolecules. They did by the anchoring sulfobutyl or amine functional groups to the PVA backbone, it is simple to generate surfaces with either positively or negatively charged surface characteristics. This grafting procedure boosted hydrophilicity, which in turn improved the ability of biomolecules to transport more weight (Jung et al., 2001). Eldin et al. (2015) used the “grafting to” approach to create chitosan (CS) grafted alginate hydrogel by using coupling agent P-benzoquinone, and hydrogel, it was created by clicking chitosan chains onto NH_2_ groups (Cai et al., 2005; Eldin et al., 2015). Cai et al. (2005) used irradiation for manufacturing hydrogels from chitosan grafted with poly(N-isopropyl acrylamide) (PNIPAAm). The swelling behavior among these chitosan-g-PNIPAAm hydrogels was improved as the proportion of grafted branches, or the volume of grafted branches, increased (Hu et al., 2002). Recently, numerous uses of chitosan grafted with acrylic acid has been find as a potential technique for producing hydrophilic and bioadhesive polymers. Hu et al. (2002) studied about the CS-poly(acrylic acid) (PAA) graft by using silk peptide as a model drug carrier for the delivery of hydrophilic medicines, proteins, and peptides (Vihola et al., 2008; Kumar and Sharma, 2013). In different research the Vihola et al. (2008) created thermostable systems by grafting poly (N-vinyl caprolactam) (PVCL) with poly (ethylene oxide) to sustain thermally responsive particles, for giving rise to PVCL-graft-C11EO42. These novel polymeric materials contained heat-resistant hydrogel particles that could be used to control medicinal release (Pistel et al., 2001; Mundargi et al., 2007b; Kulkarni and Sa, 2008). In a similar manner the transdermal films were created by Mundargi et al. (2007b) by utilizing tailored XG, which was created by grafting acrylamide to XG. These customized XG films successfully achieved a controlled release of atenolol drug. Siraj et al. (2014) combined PVAL with 6-thioguanine and methylcellulose (MC), for producing an interpenetrating polymer network microsphere that successfully regulated the release of the medication in the *in-vivo* testing (Kumbar et al., 2003).

The pain modulator drug Indomethacin was blend with thepolyacrylamide-grafted chitosan microspheres, and crosslinked with glutaraldehyde for treating arthritis by Kumbar and group The indomethacin was first released from such microspheres through a polymer chain relaxation, but with time controlled molecular diffusion was the primarily responsible for the release from the completely inflated polymer (Yoshioka et al., 1995).

Some additional polymeric grafted systems, like chitosan grafted microspheres, were made by using highly crosslinked glutaraldehyde for encapsulation of the antihypertensive medication nifedipine, which was examined to obtain site-specific drug delivery system. Liu et al. (2001) created the *N*-dodecylated chitosan for intracellular delivery of gene therapy. Additionally, the complex was demonstrated to improve activity and shield DNA against DNA nuclease (Nam et al., 2015). Polymeric micelles were synthesized by Nam et al. from two medications grafted onto chitosan. O-carboxymethyl chitosan was first coupled with tocopherol to generate tocopherol *O*-carboxymethyl (TOC), and then HP-TOC-DOX polymeric micelles were made by attaching doxorubicin and an anti-human epidermal growth factor receptor 2 (HER2) target peptide. The anti-HER targeting peptide was used to enhance the cellular uptake and therapeutic effectiveness, which was demonstrated through *in- vivo* investigation (Chen et al., 2016b).

## 6 Recent advancements in polymer grafting technique

### 6.1 Photopolymerization

**FIGURE 17:**
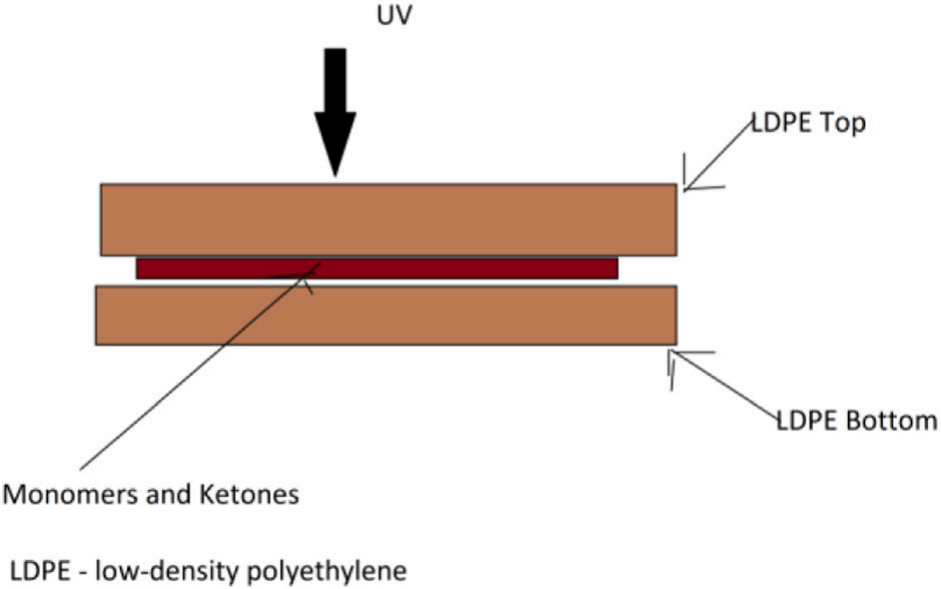
Photo-polymerization method set up of interlayer films.

The use of photochemistry is one of the novel approaches that has widened the scope of bio-conjugation. The field of biochemistry has relied heavily on the light chemistry for many years since its first ever use. Photo-affinity labeling (a method for identifying active and binding sites) and photodynamic treatment were two of the first areas where it was successfully used with tiny molecules. Recently, it has been clear that light may be used to aid the synthesis of well-controlled polymers under very mild circumstances, which is a major advantage for grafting from polymerization systems (Goldmann et al., 2015). Light also allows the temporal and spatial manipulation of the polymer. The benefits of grafting-to conjugations have been used to create sophisticated three-dimensional settings for cell growth *via* using three common techniques such as 1) photo-patterning, 2) the exact positioning of polymers, 3) the development of novel biomaterials (Fu et al., 2018). There is still a lot of room for research, development, and improvement in the field of using light to accomplish polymer–protein bioconjugation in the near future (Figure 17).

More sophisticated systems, such as living cells, are being explored, to direct conjugate with the wider range of amino acids under more bio-friendly circumstances (Chatani et al., 2014). The chromophore (a part of photocatalyst that absorbs light) triggers photo-polymerizations through electronic transition (excitation) by the exposure of light in the chromophore. This exciting chromophore can dissipate its excess energy by emitting heat or light, breaking a homolytic bond, or transferring energy or electrons to another molecule. Polymerization can be initiated by radicals produced *via* homolytic bond cleavage or energy/electron transfer to another moiety.

To quantify this process, the quantum yield helps to quantify the photo polymerizations since it determines the likelihood of a certain photochemical process. Several varieties of artificial light sources are available in the market and have been used for photopolymerization such as fluorescent lights, light-emitting diodes, and lasers are the most widely used alternatives. Although fluorescent lights are low-priced and easy to install, and, their wide emission profiles make them less efficient than alternative lighting options. The demerit of laser light is its higher cost, so they are not widely used for photopolymerization, despite their monochromatic emission and accuracy. LEDs are a new kind of lighting that has gained popularity owing to their favorable characteristics, including their narrow emission profile, cheap price, simple design, and high efficiency. LEDs may also produce light beyond the visible area of the electromagnetic spectrum, which expands their usefulness (Zhu et al., 2013).

### 6.2 Surface grafting polymer

**FIGURE 18:**
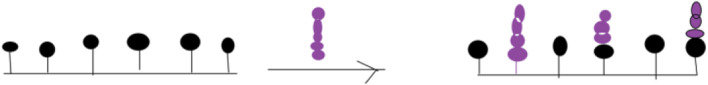
General method of surface grafting.

Surface-grafting, a kind of surface modification that has gained attention in the field of organic electronics due to its distinctive characteristics. Over the course of the previous several decades, different surface grafting polymers have proven to be effective as insulating layers, conductive layers, and semiconducting layers in a wide variety of devices to improve their electrical performance (Wang et al., 2020). Due to the rapid advancement of flexible electronics, the surface-grafting polymers possess inherent flexibility and environmental endurance, which have a high possible function value in flexible devices. Surface grafting polymers have many advantages over other surface processing methods. Surface grafting, in which specific-function polymers are grafted to the substrate’s surface, moreover, it is a proficient method for adjusting the surface energy (Figure 18) (Chen et al., 2012). Surface grafting techniques also useful for the modification of polymer brush thickness and grafting density by optimizing critical parameter, involving the process.

For instance, the high-density insulating polymer brushes helps to decrease the leakage current of polymer layer pinholes compared to the traditional techniques (spin coating, scrape coating, inkjet printing, etc.). Surface grafting is also a practical technique for increasing charge injection by tuning the interface’s energy. The second advantage is practical patterning, which involves high-resolution patterning techniques like photolithography and electron beam lithography that might be used with polymer brushes. Some other advantageous of surface grafting are following,

(I) It is quite steady, because of its chemical interaction with the substrate. It is mostly used in the production of flexible devices, and they possess superior durability for organic solvents and mechanical resilience against stripping is a boon.
(II) It has the highest degree of congruence. Polymer brushes can be stuck to any surface, no matter how smooth or rough it is. The organic active layers of the devices are more compatible with the surface-grafting polymers than with their inorganic counterparts (Lau et al., 2012; Welch and Ober, 2013).

As the field of organic electronics expanded rapidly, the surface-grafting polymers was also expanded as promising building blocks for a broader range of organic devices, counting organic field-effect transistors (OFETs), organic light-emitting diodes (OLEDs), and organic photovoltaics (OPVs) (Biniek et al., 2020). The “grafting to” method involves the modification of the substrate with a chemical group, followed by the grafting of readymade polymer onto the surface by a controlled chemical reaction between the chemical group and the terminal group of the preformed polymer chain. In particular, the “grafting to” technique does not include a monomer polymerization event during the production of polymer brushes, but often depends on a chemical coupling or condensation reaction between the end group of the preformed polymer and the surface group of the substrate. For example, the grafting of polystyrene (PS) from two unique “grafting to” strategies available for making PS brushes: The first is a condensation reaction between polystyrene (PS-OH) with terminal hydroxyl groups and silicon dioxide (SiO_2_), and the second is a coupling reaction between polystyrene terminated in dimethylchlorosilane (PS-Si(CH_3_)_2_Cl) and a surface that has been rendered hydroxyl-functionalized through the use of ultraviolet (UV) ozone or oxygen plasma cleaning. These two methods of “grafting to” have the potential to form stable covalent connections with the substrate (Zhao and Brittain, 2000; Tsujii et al., 2006; Wang et al., 2010; Liu et al., 2011). The siloxane groups of several polymers, such as PFS37, PHIC-b-PTMSM38, and poly(3-hexylthiophene-), were tried to couple with hydroxylated substrates to form polymer brushes The controlling polymer-surface interactions with random copolymer brushes was initially described by Hawker et al. The end-functionalized copolymers were grafted over the silicon substrates with a thickness of around 5 nm, with a fast and easy process. However, the “grafting to” method makes it difficult to construct high-density polymer brushes due to the steric effect, which occurs when one polymer chain prevents the attachment of another polymer chain to a surface. The aforementioned limitations are overcome by the grafting from approach, which is now widely used to make high-density polymer brushes by surface-initiated polymerization and *in-situ* reaction (Dugas et al., 2010; Chen et al., 2017). The “grafting from” method involves securely attachment with the initiator to the surface of the substrate by certain chemical processes such as chemical coupling, condensation reaction, etc. (Liu et al., 2004). Then, once a precursor solution is added, the polymer chain grows in length from the surface, and creates dense polymer bristles. Several “grafting from” methods are currently used in organic electronics, such as surface chemical oxidation polymerization (SCOP) (Jordan, 2006), surface-initiated polymerization (SIP) (Khabibullin et al., 2015) (including surface-initiated atom transfer radical polymerization (SI-ATRP) (Zhang et al., 2014), surface-initiated ring-opening metathesis polymerization (SI-ROMP) (Ohno et al., 2011), and surface-initiated reversible-addition fragmentation chain transfer (SI-RAFT) (Zhou, 2015). In SI- ATRP, the halogen atoms are transferred from inactive species to catalysts through electron transfer and atom transfer, leading to the formation of active radicals and metal complexes in a more oxidized state. It is able to polymerize quickly and easily at room temperature, by giving little stress on the polymer. SI-RAFT differs from other SIP methods, because of the use of a chain transfer agent (CTA) to regulate polymerization. CTA or traditional free radical initiators are often grafted onto the substrate to provide anchoring sites, because radical transfer occurs continuously as chains grow, it may controlled throughout the polymerization process. CTA attachment preceded electro deposition and SI-RAFT for the growth of poly(N-vinyl carbazole) PVK brushes on ITO.

The conventional chemical oxidation polymerization used to produce surface-grafting polymers by attachment of initiator, followed by the oxidative polymerization of monomers. The surface chemical oxidation polymerization (SCOP) method with FeCl_3_ as the oxidant, was used to utilized to regulate the growth of conductive PPy brushes. Multiple advantages from the “grafting from approach makes this technique preferable over the “grafting to” method, which limits its applicability to organic electronics like adjusting the reaction parameters, like the concentration of the grafting agent, the time of the reaction, etc., allows for precise control over the density and thickness of grafted polymer films. The exact patterning of the initiator using lithographic methods that may result from the “grafting from” approach is useful for fabricating devices across wide areas (Herzer et al., 2010; Bousquet et al., 2014).

### 6.3 Conducting polymers

The fourth generation of polymeric resources is comprised of conducting polymers. For decades, the physicochemical features hereditary from conventional polymeric materials has been improved remarkably for their electrical conductivity, for that metals have placed this class of materials at the forefront of energy-related scientific study. Conducting polymers seem to become increasingly appealing in a variety of biomedical uses, as well as energy applications centered on electrical and electronic characteristics, because of their functional chemistry in the presence of electrical fields from numerous kinds of tissues such as muscle, connective tissue, epithelium, and nervous tissue. In the 1980s, the possibility of synthesizing novel conducting polymers with improved and preferred characteristics started to draw the interest of synthetic chemists. Since then, several conducting polymers and their derivatives have been manufactured and utilized for their causes (Maity and Dawn, 2020).

The occurrence of conjugated double bonds throughout the backbone of a conducting polymer is a significant and distinctive characteristic. However, conjugation by itself is insufficient to render a polymer conductive (Guimard et al., 2007). The conductive material requires for the addition of electrons or holes, so that polymer can be loaded with extra charge carriers, so that It can be useful to introduce carriers into the electron orbitals; it also induces carrier delocalization along the polymer chain and charge carrier mobility, which is extended to three dimensions through interchain electron transfer. An organic polymer, typically an insulator or semiconductor with low conductivity in the range of 1010105 S cm^1^, is transformed into a polymer with a “metallic” conducting domain even during the doping process (about 1–104 S cm^−1^) (Wan, 2008).

The physicochemical characteristics of a conducting polymer are affected by numerous factors, including the chemical identification of the monomer, the molecular weight, the presence of functional groups and side chains attached to the monomeric units, and even polymer form (Smela, 2003). In spite of that the all variables that impact electron delocalization next to the major chain have a direct action on the polymer’s conductivity. Therefore, creating a conducting polymer requires, far more than simply defeating synthetic difficulty. Occasionally, the functionalization of conducting polymers adds difficulty compared to the synthesis of the parent molecules. In the realm of conducting polymer-based technology development, processability, morphology, stability, and durability are among the most important factors (Ramanavicius and Ramanavicius, 2021). These qualities are not necessarily completely known by the chemical composition of the backbone but, still also by additional variables, like the accessibility of functional groups, the side chain’s composition, etc. As a result, changing the chemical structure while keeping the polymeric backbone intact is a well-known technique for fine-tuning the physicochemical properties of conducting polymers. The side chains of conjugated polymers are primarily used as solubilizing groups. However, the overall contribution of side chains is substantial, and a mutation in a side chain can have a direct impact on optical, electronic, structural, and electrical properties. As a result, the side chain functionalization of conducting polymers has become a popular method for fine-tuning polymer characteristics. The polarity of the side chain (electron-drawing vs. electron-donating) influences the charge transport along with the main chain, however, the steric factor of the functional group changes the planarity and conformation of the main chain, which impacts on conjugation. However, the alteration of side chains, reduces the conductivity of the original polymer (Jaiswal and Menon, 2006; Kroon et al., 2017).

The blending of copolymer with another polymer or by grafting, can modify the physicochemical characteristics of a polymer even more dramatically. As an alternative to lithography, conducting polymers as constituents of block-copolymer systems has been described for nanostructural control (Zhang et al., 2018). As a result of the diluting impact of non-conducting components, the conductivity of this material might be drastically diminished. Polymer grafting (Opsteen and van Hest, 2005; Li et al., 2017) is an ancient and traditional technique specially in the case of conducting polymer, which remains in advancement. Conducting polymer grafting is critical because it is not changing the primary chain’s elongated conjugated structure but also can introduce and integrate the properties of the grafted components. Grafting can compensate for and increase the characteristics of conducting polymers followed by charge transfer, so it can be further corelated to solubility, nano-dimensional shape, biocompatibility, bio-communication, etc. For qualifying the technical or biological applications, conducting polymers require numerous additional criteria in addition to electron delocalization (Dubey et al., 2020).

### 6.4 Conductive polymer covalent grafting

The standard technique for the manufacture of graft copolymers is the covalent approach. It ensures that the characteristics of similar species are strongly blended and, as a result, allows for the design of materials to meet specific needs. Khairkar and Raut (2014) created chitosan-graft-polyaniline by oxidative polymerizing aniline in an acidic environment with ammonium persulfate (APS) as a catalyst. This method was reported with high yield an ecologically friendly, cost-effective conductive biomaterial for sensing applications Moradi and Rezaei (2022) developed a new kind of adhesive by fusing novolacs to polyaniline (PANI), in that the aniline was polymerized to produce the final graft polymer after novolacs were grafted with p-aminobenzoic acid. In the result the polymer, which was used a conductive glue, showed a drop in tensile strength and flexibility after grafting, because of the unaltered behavior of PANI’s conductivity (Moradi and Rezaei, 2022).

### 6.5 Non-covalent polymer conductor grafting

Covalent binding to the polymer backbone is the common definition of grafting however, covalent grafting have downsides in some circumstances like the complex synthetic structure, unwanted characteristics modification, and the irreversible character of connectors are just a few of the key problems that have prompted chemists to consider a more practical approach. In view of that the non-covalent imerges with these implications, through an unconventional grafting approach. The non-covalent blending was considered as supramolecular grafting, because of its significance and potential applications in the field of conducting polymers (Wang et al., 2015). The direct connection to the conducting polymer backbone, have an adverse action on main chain conjugation, but grafting may provide the required durability and ease of processing in addition to its unrivaled electrical and electronic functionality. Geng et al. (1999) used phosphonic acid with a hydrophilic tail to dope polyaniline as one of the first examples of making soluble polyaniline (PANI). The doped polymer was soluble in water, NMP, and chloroform when the 550-Mw hydrophilic chain of poly(ethylene glycol) monomethyl ether (PEGME) was used as the dopant. The Films was used to pour the wide variety of solutions, including water, chloroform, and NMP. The electrochemical reversibility of the film in the aqueous medium was exceptional with the higher rate of conductance. The DSC spectra showed reversible phase transitions at 19.1°C for the cooling curve and 9.8°C for the heating curve, likely due to the melting and crystallization of hydrophilic PEGME segments. In this system architecture, the combination of two polymeric components occurs *via* ionic addition (Geng et al., 1999). The self-assembled property of the same composite system was later investigated by Nandan et al., and found that the strong repulsion between the PANI backbone and the PEG side chain led to a microphase-separating lamellar morphology consisting of alternating ionic layers containing PANI backbones and the ionic head groups of the dopant, and non-ionic layers containing the PEG side chain. It was found that whenever the thickness of the non-ionic layer increased, the chain stretched because of the greater distance between the PANI backbone connection locations. Temperature-dependent SAXS analysis found that the compound underwent an order-to-disorder transition due to de-protonation at about 225°C. The conformational stiffness of the PANI backbone and the strong ionic link between the backbone and the PEG side chains significantly slowed the crystallization kinetics and crystallizability of the PEG side chains in the structures. Changes in behavior were more pronounced in the conformationally flexible PEG segment than in the conformationally rigid PANI backbone (Nandan et al., 2004; Arslan, 2012).

## 7 Patents in area of polymer grafting

Some of the patents are given below Table 3, with the summary of the inventions.

**TABLE 3:** Patents involving polymer grafting.

S. No.	Patent publication number	Summary of the invention	References
1.	US 20040208931 A1	Two polymers were used to create the dosage form, with the first polymer serving as the deposit and containing the active ingredient, and the other one, a polyvinyl alcohol-polyethylene glycol graft copolymer, serving as the cover layer (PVA-PEG)	Chen et al. (2005)
2.	US 7419685 B2	In this case, solid dosage forms are created using a water-swellable graft copolymer or a combination of graft copolymers as a polymeric binder.	Mundargi et al. (2007a)
3.	WO 2007115381 A2	It entailed employing PVA-PEG copolymers, such as Kollicoat IR, to make solid dispersions of bioactive chemicals having low water solubility and dissolution rates.	Silva et al. (2009)
4.	US 17/243,133	The synthesis and applications of an ofloxacin-imprinted polymer with an AM-type polystyrene microsphere are covered by this patent. Current technology can considerably improve a drug’s efficacy against Gram-ve and +ve bacteria, with S-antibacterial ofloxacin being 8 to 128 times more effective than its enantiomer R-ofloxacin.	Toti and Aminabhavi (2004)
5.	US 14/548,383	In this invention, a device with an enhanced porosity membrane was described. The modified porous membrane is a porous membrane that has a polymer coating grafted onto it. The device is used to identify analytes using immunoassays in biological samples.	Sutar et al. (2008)
6.	US 16/479,583	In this patent, a reconfigured alginate copolymer with an alginate spine and a grafted substituent attached to one of the alginate backbone’s hydroxyl groups was described.	Mundargi et al. (2007b)
7.	US 6,384,110	This patent described a unique graft copolymer that is created by combining different monomers, such as methyl methacrylate and styrene, with an oil-modified polyurethane resin.	Kulkarni and Sa (2009)

## 8 Natural polymers grafting

The various examples of natural based polymer grafting and its used is summarized in the below given Table 4, which was the best way to utilize and altered the specific property of natural polymer, specifically in the targeted delivery of the active drug.

**TABLE 4:** Natural grafted polymers.

S.No.	Natural polymers (gum used as backbone)	Grafting technique	Applications	References
1	Acacia	By chemical modification means	Used as disintegrating agent, osmotic delivery of drug, dental plaque	Trivedi et al. (1986)
2	Cashew	Free radical polymerization	Sustain drug delivery	da Silva et al. (2007) Kumar et al. (2012)
3	Gellan	Microwave based grafting	Sustain drug delivery, ophthalmic delivery, hydrogels preparation, lowering cholesterol and, thickening agent	Deogade et al. (2012) Gandhi et al. (2019)
4	Okra	By chemical modification means	Controlled drug delivery, as binding agent, for better Digestion, bone strengthen, vision, cancer treatment (lung and oral)	Mishra et al. (2008) Sah (2016)
5	Guar	By chemical modification means	Disintegrating agent, film coating purpose,	Chaurasia et al. (2006) Singh et al. (2014)
6	Sesbania	By chemical modification means	Used as gelling agent	Stehling et al. (1998)
7	Karaya	Heat method	Muco and buccal adhesive, gelling agent, treating diarrhea, laxative	Gandhi et al. (2019)
8	Xanthum gum	By chemical modification means and thermal method	Lowering cholesterol, In the treatment of cancer, ulcerative colitis	Vendruscolo et al. (2005)

## 9 Future prospect

The modification of the polymer through grafting will lead the application of the polymer to the different field not only the pharmaceutical but also to the electrical and related field. The special concern is related to the ecologically friendly, cost-effective conductive biomaterial which is a need in the current scenario. The surface modification of the polymer for the biodegradable substance is still challenge, as the use of plastic/polyethene in the packaging industry is increasing day by day, however the modification is still in progress. The biodegradable material/polymer will be the future of plastic industry, if the grafting technic can enhance the biodegradable nature with similar strength. The conducting polymers can be used in the electrification of the automobile industry can reduce the burden of petrochemicals. In spite of various challenges in the polymer grafting technology, the filed is emerging vastly and will replace all the polymer with more advanced grafted polymer with more effectiveness.

## 10 Conclusion

The graft polymerization is an important procedure that enhances the advanced characteristics of the backbone polymer. This method has great potential to improve the characteristics of polymers like charge transport capability which results to solubility, nano-dimensional characters, biocompatibility, biocommunication, and so on. The results of the change in the structure and chemistry of numerous polymerization reactions are crucial for developing and broadening the multifunctional applications of a particular polymer.

The grafting of parent polymer can alter the desired properties of natural/synthetic polymers without changing their basic character comes under the polymer grafting, among them click reaction is a widely utilized method for graft polymerization and receiving great attention due to its easy approach and procedure. The effectiveness of the graft-to approach is mainly dependent on the coupling chemistry used, the behavior of the chain side, and the length of the chain. Polymer grafting is a helpful technique for creating supplies with unique properties. Graft copolymerization is an efficient way of adding beneficial applications to the major polymer backbone, which is utilized in a variety of uses. Grafting synthetic polymers is an easy method for the addition of new characteristics to a natural polymer while retaining the original characteristics of the substrate. Grafted copolymers are crucial in modifying their physicochemical characteristics. Grafted/cross-linked copolymer is an effective and novel method with various applications including drug delivery, adsorption, textile/tannery wastewater treatment, domestic/sewage wastewater treatment, agriculture, and humanitarian service.
